# Accuracy and reliability of noninvasive stroke volume monitoring via ECG-gated 3D electrical impedance tomography in healthy volunteers

**DOI:** 10.1371/journal.pone.0191870

**Published:** 2018-01-26

**Authors:** Fabian Braun, Martin Proença, Andy Adler, Thomas Riedel, Jean-Philippe Thiran, Josep Solà

**Affiliations:** 1 Systems Division, Centre Suisse d’Electronique et de Microtechnique (CSEM), CH-2002 Neuchâtel, Switzerland; 2 Signal Processing Laboratory (LTS5), Ecole Polytechnique Fédérale de Lausanne (EPFL), CH-1015 Lausanne, Switzerland; 3 Systems and Computer Engineering, Carleton University, Ottawa, ON K1S 5B6, Canada; 4 Cantonal Hospital Graubuenden, CH-7000 Chur, Switzerland; 5 University Children’s Hospital and University of Bern, CH-3010 Bern, Switzerland; 6 Department of Radiology, University Hospital Center (CHUV) and University of Lausanne (UNIL), CH-1011 Lausanne, Switzerland; Worcester Polytechnic Institute, UNITED STATES

## Abstract

Cardiac output (CO) and stroke volume (SV) are parameters of key clinical interest. Many techniques exist to measure CO and SV, but are either invasive or insufficiently accurate in clinical settings. Electrical impedance tomography (EIT) has been suggested as a noninvasive measure of SV, but inconsistent results have been reported. Our goal is to determine the accuracy and reliability of EIT-based SV measurements, and whether advanced image reconstruction approaches can help to improve the estimates. Data were collected on ten healthy volunteers undergoing postural changes and exercise. To overcome the sensitivity to heart displacement and thorax morphology reported in previous work, we used a 3D EIT configuration with 2 planes of 16 electrodes and subject-specific reconstruction models. Various EIT-derived SV estimates were compared to reference measurements derived from the oxygen uptake. Results revealed a dramatic impact of posture on the EIT images. Therefore, the analysis was restricted to measurements in supine position under controlled conditions (low noise and stable heart and lung regions). In these measurements, amplitudes of impedance changes in the heart and lung regions could successfully be derived from EIT using ECG gating. However, despite a subject-specific calibration the heart-related estimates showed an error of 0.0 ± 15.2 mL for absolute SV estimation. For trending of relative SV changes, a concordance rate of 80.9% and an angular error of −1.0 ± 23.0° were obtained. These performances are insufficient for most clinical uses. Similar conclusions were derived from lung-related estimates. Our findings indicate that the key difficulty in EIT-based SV monitoring is that purely amplitude-based features are strongly influenced by other factors (such as posture, electrode contact impedance and lung or heart conductivity). All the data of the present study are made publicly available for further investigations.

## Introduction

The measurement of central hemodynamic parameters is of importance to manage critically ill patients. An example are patients who undergo high-risk surgical procedures. In this group of patients, the continuous post-surgical monitoring and early hemodynamic optimization has shown to be very important as it results in significantly reduced mortality [1, 2]. The goal of hemodynamic optimization is to ensure an adequate tissue perfusion and organ function. This is achieved by continuous monitoring and manipulation of hemodynamic parameters (e.g. SV, CO and oxygen saturation) via therapeutic interventions (including fluid challenge or drug administration). Two central hemodynamic parameters of importance are the cardiac output (CO) and the related stroke volume (SV), since they are closely linked with oxygen delivery and the health of the heart. However, right heart thermodilution, which is considered as the clinical reference method for CO measurement, requires highly invasive catheterization and is known to cause complications without decreasing mortality [3]. Even though less invasive and noninvasive measurement techniques are available [4, 5, 6], they do not fulfill the requirements of an “ideal” hemodynamic monitoring device as defined by Vincent et al. [7]. A recent meta-analysis of noninvasive CO monitoring devices by Joosten et al. [8] has found that none of these devices is able to provide accurate enough measurements in clinical settings. Therefore, the quest for the “ideal” CO monitoring devices continues. A potential technology is electrical impedance tomography (EIT), which has been investigated in previous studies as a low-cost and radiation-free medical imaging modality for the noninvasive and continuous monitoring of SV [9, 10, 11, 12].

In brief, EIT consists of a belt of electrodes applied around the thorax, which measures electrical impedances by injecting weak alternating currents [13, 14, 15]. These measurements are transformed into tomographic images which represent changes in intra-thoracic impedance. EIT is commonly used to monitor lung function in order to optimize regional ventilation or to diagnose lung diseases [13]. In contrast, the EIT-based assessment of cardiovascular activity is at an earlier stage of research [16]. The few studies published, which address the estimation of SV and CO via EIT [9, 10, 11, 12] are described hereafter and all raise the need for further investigations.

The EIT-based SV measurement was first reported by Vonk Noordegraaf et al. [9] in 2000. In 23 patients and 11 healthy volunteers, they derived absolute SV values from the amplitude of the temporal signal in the heart region and the duration of a cardiac cycle. In 2014 Pikkemaat et al. [10] investigated the feasibility of estimating SV in 14 pigs via the heart-related impedance change by using a subject-specific one-point calibration. In certain animals their measurement was impaired by an unknown scaling of the heart amplitude, which they related to variations in lung volume and also to craniocaudal displacement of the heart with respect to the EIT electrode plane. While not available in the corresponding publication [10], in his thesis [11], Pikkemaat also reports on the analysis of the lung-related impedance change $z^{{\text{SV}}_{p}}$. In experiments where SV was modulated by changing the ventilation settings (the positive end-expiratory pressure—PEEP), $z^{{\text{SV}}_{p}}$ showed a higher correlation with SV (*r* = 0.69) when compared to the heart-related impedance change $z^{{\text{SV}}_{c}}$ (*r* = 0.64). On the other hand, when SV was modified using dobutamine (a positive inotropic agent leading to a SV increase) $z^{{\text{SV}}_{p}}$ correlated less with SV (*r* = 0.61) than $z^{{\text{SV}}_{c}}$ (*r* = 0.64). In contrast, in the very recent study by da Silva Ramos et al. [12] EIT-based SV was successfully estimated from the systolic amplitude in the lung region. In this study, large variations in SV were induced via hemorrhagic shock and subsequent fluid challenges in twelve pigs. While they could show an acceptable trending ability with 91.2% concordance rate, their absolute SV measurements were only accurate when taking into account body dimensions (i.e. a subject-specific calibration).

The abovementioned studies show contradictory outcomes (for EIT-based SV estimation via the impedance changes in both the heart and lung region) and require a detailed investigation of this approach in human subjects. To this end, we performed a study on ten healthy volunteers undergoing an experimental protocol resulting in large variations of SV. These variations were estimated using EIT and compared to noninvasive SV reference measurements derived from the oxygen uptake ${\dot{V}}_{O_{2}}$. In addition, to reduce undesirable influences of the abovementioned craniocaudal heart displacement and to assure accurate image reconstruction [17, 18] an improved EIT measurement setup was used. This setup consists of ECG-gated 3D EIT in combination with an individual—subject-specific—reconstruction model generated by means of a commodity 3D camera.

## Methods

### Study protocol and study population

Ten healthy adult volunteers (9 male/1 female, weight: 68.9 ± 10.8 kg, height: 179.3 ± 8.2 cm, BMI: 21.3 ± 2.0 kg/m^2^, age: 35.4 ± 4.1 years) were enrolled in the study, of which all provided written informed consent. The study was approved by the local ethics committee of the canton of Vaud, Switzerland (CER-VD, 2017-00709).

**Fig 1:**
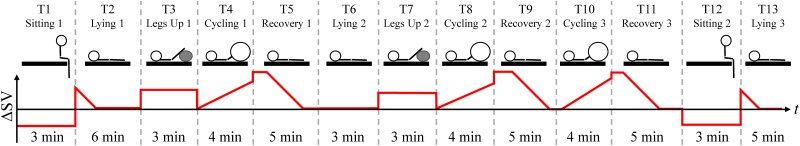
Temporal evolution of the experimental protocol consisting of the thirteen tasks (T1 to T13) illustrated on top and the expected changes in SV shown below. The protocol comprises different postures such as sitting (T1 and T12), lying in supine position (T2, T6 and T13), lying with legs up (T3 and T7), cycling in supine position (T4, T8 and T10) and the subsequent recovery periods (T5, T9 and T11).

This study was performed in the physiology laboratory facilities at the Swiss Center of Electronics and Microtechnique (CSEM, Neuchâtel, Switzerland). There, the subjects underwent an experimental protocol during approximately one hour including postural changes (lying flat, lying with legs up, and sitting) and bicycle exercises (cycling in supine position). The thirteen tasks (T1 to T13) performed were expected to lead to SV variations as illustrated in Fig 1 and described hereafter. These SV variations are considered with respect to the baseline SV level at the end of the three lying positions (T2, T6 and T13). While sitting (T1 and T12) a lower SV is expected due to a decrease in cardiac preload. Similarly, while lying with legs up (T3 and T7), a higher SV is expected. Moreover, after the transition from sitting to lying (T1 to T2 and T12 to T13) a sharp increase of SV and subsequent decay to baseline is expected due to the augmentation in cardiac preload caused by the sudden increase in central venous return [19]. Finally, the cycling exercises in supine position (T4, T8 and T10) are expected to increase the SV, with a further—but temporary—augmentation during recovery (T5, T9 and T11) followed by a steady decrease (as reported by Cumming [20] and also known for upright exercise [21]).

### Data acquisition

**Fig 2:**
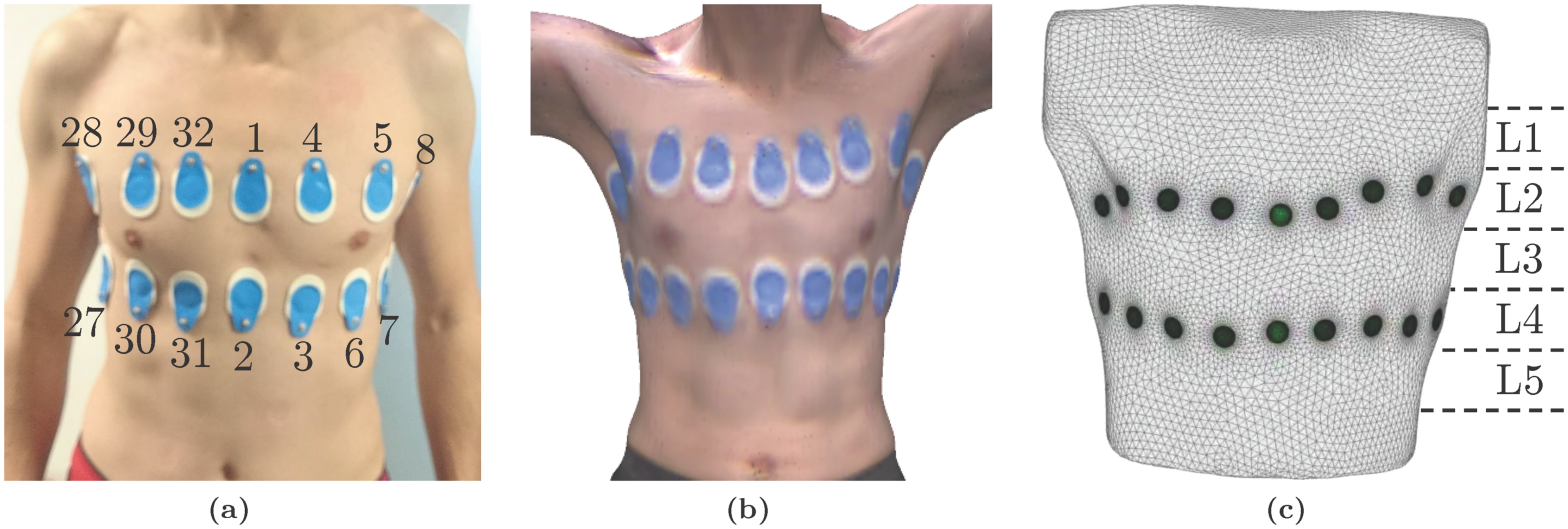
(a) Placement of the 32 gel electrodes used for EIT: two planes of 16 electrodes each are placed above and below the nipple line. (b) Example image of the 3D camera and (c) the resulting 3D subject-specific model of the thorax including the electrodes (green circles). L1 to L5 illustrate the five planes on which EIT data was reconstructed.

First, the volunteers were equipped with 32 self-adhesive gel electrodes (BlueSensor T-00-S, AMBU, Ballerup, Denmark), placed on two planes with 16 electrodes each: one above and one below the nipple line, as shown in Fig 2a. Second, to obtain a subject-specific anatomical model and the correct electrode positions, the 3D surface of the subject’s thorax was acquired using a dedicated software (ReconstructMe, version 2.5.1034, PROFACTOR GmbH, Steyr-Gleink, Austria) in combination with a 3D camera (Kinect XBOX 360, Microsoft, Redmond, USA). An example of such a 3D image is shown in Fig 2b. Then, the 32 electrodes were connected to a slightly modified version of the EIT SensorBelt (Swisstom AG, Landquart, Switzerland) [22] in combination with the BB^2^ EIT device (Swisstom AG, Landquart, Switzerland). To achieve this connection, the conductive textile was disconnected from the active electrodes and instead, commercially available ECG cables were attached and connected to the self-adhesive gel electrodes. The electrodes were arranged as shown in Fig 2a, which results in the use of the “square pattern with skip 4” as suggested by Grychtol et al. [23] for 3D EIT.

**Fig 3:**
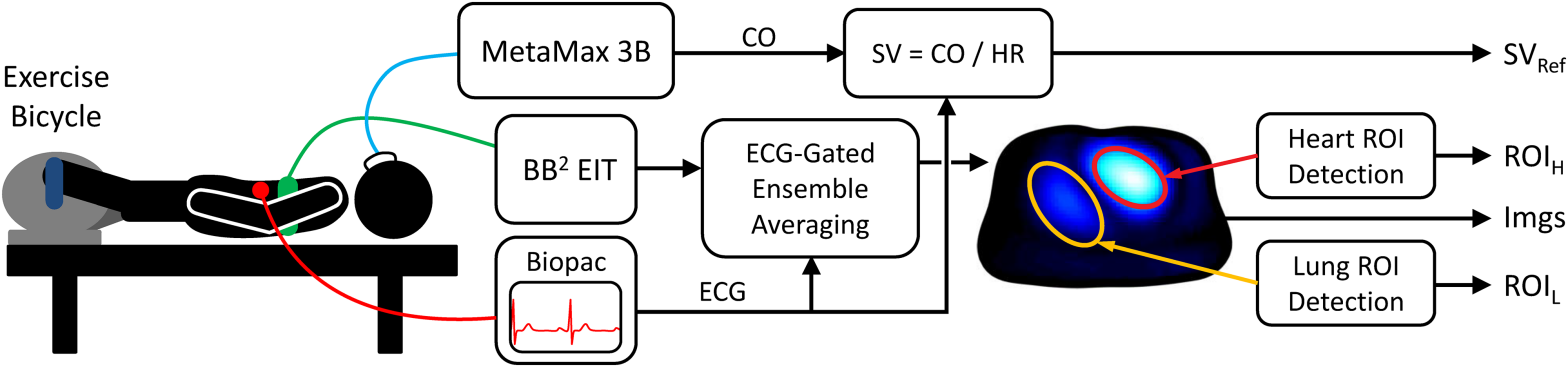
Block diagram of the measurement setup and the first processing steps resulting in ECG-gated EIT images (Imgs) and the regions of heart (ROI_H_) and lung (ROI_L_).

An ECG was recorded using the ECG100C module (Biopac Systems, Inc., Goleta, USA). Furthermore, CO reference measurements were performed via the oxygen uptake ${\dot{V}}_{O_{2}}$ and the method described by Stringer et al. [24] using MetaMax 3B (CORTEX Biophysik GmbH, Leipzig, Germany). To this end, a mask was placed on the subject’s face to measure air flow and gas exchange. This measurement setup is also illustrated in Fig 3.

### Data preprocessing

First, EIT and hemodynamic data were manually aligned in the time domain with the help of deliberate spikes induced via synchronous tapping on EIT and ECG electrodes at the beginning and at the end of each recording. Then EIT samples were interpolated in the time domain to correct for the sporadic loss of certain EIT frames. Furthermore, a clock drift between EIT and ECG signals of around 0.1 s/h was observed and corrected for.

As also illustrated in Fig 3, EIT data was averaged via ECG-gated ensemble averaging [25, chap. 3.5.1] to one representative cardiac cycle per measurement. To do so, all data were first low-pass filtered (4^th^-order Butterworth with *f*_*c*_ = 6.5 Hz), then high-pass filtered (4^th^-order Butterworth with *f*_*c*_ = 0.75 ⋅ HR/60, with HR as the current heart rate), and finally aligned to the ECG’s R-peaks. To this end, the measurements of each of the thirteen tasks (T1 to T13 in Fig 1) were split into one-minute sequences and each sequence was averaged to one cardiac cycle as mentioned above. Due to strong movement artefacts, data from the cycling exercises (T4, T8 and T10) were excluded from analysis.

Besides, the continuous CO measurements were divided by the instantaneous HR and averaged in the same one-minute intervals to obtain SV reference values SV_Ref_. It has to be noted that the CO reference device (MetaMax 3B) does only provide absolute CO values (in L/min) if the maximal oxygen uptake (${\dot{V}}_{O_{2}-\text{max}}$) is known for each subject, i.e. $\text{CO}={\dot{V}}_{O_{2}}/(57.21+104.7\frac{{\dot{V}}_{O_{2}}}{{\dot{V}}_{O_{2}-\text{max}}})$ [24]. Since ${\dot{V}}_{O_{2}-\text{max}}$ was not evaluated in the present experimental protocol, it was estimated using the model suggested by Jackson et al. [26]:

$$\begin{array}{c} {\dot{V}}_{O_{2}-\text{max}}=(56.363+1.921\cdot \text{PAS}-0.381\cdot A-0.754\cdot \text{BMI}+10.987\cdot S)\frac{W}{1000}\;\left[\text{L}/\text{min}\right] \end{array}$$ (1)

Where PAS denotes the physical activity on the NASA/JSC scale [26], A the age in years, BMI the body mass index in kg/m^2^, S the sex (0 female, 1 male), and W the weight in kg.

### Subject-specific EIT image reconstruction

For each volunteer, a subject-specific model for EIT image reconstruction was created. To do so, the 3D surface of the thorax scan (acquired as described before and shown in Fig 2b) was processed in Blender (version 2.78c, Blender Foundation, Amsterdam, the Netherlands) by cropping parts not located in the EIT planes of interest (e.g. arms and neck) and transformed to a triangulated mesh. The electrode positions were then manually located in the 3D scan. The thorax mesh was further resampled and smoothed using OpenFlipper (version 3.1, Computer Graphics Group, RWTH Aachen, Germany) [27]. Finally, the electrodes were placed on the mesh using the approach proposed by Grychtol and Adler [28] and implemented in EIDORS [29]. An example of such a subject-specific thorax model is shown in Fig 2c.

EIT data were reconstructed using the 3D GREIT algorithm [23] onto images with 32 × 32 × 5 voxels. The five image planes L1 to L5 used for reconstruction (see Fig 2c) are equally spaced at a distance of half the spacing between the two electrode planes. L2 is placed at the height of the upper, L4 at the height of the lower, and L3 in between the two electrode planes. The algorithm was trained using roughly 10,000 targets located on eleven equidistantly spaced levels: at each voxel location (on the five image planes L1 to L5) plus six more planes (one located above L1, four in between L1 and L5, and one below L5). To focus image reconstruction on the three central image planes (L2 to L4) the seven target planes located in the middle contain twice as much targets than the two uppermost and lowermost target planes. To achieve a comparable noise performance (independent of the geometry of the subject’s thorax) each algorithm was set to have a fixed image SNR ($\bar{\text{SNR}}=6.5\times 10^{-3}$) [30], which compares to an average noise figure of 0.53 [31, 32].

For each subject an individual background conductivity *σ*_BG_ was used for the reconstruction model. *σ*_BG_ was obtained by finding the closest fit (in terms of absolute error) between simulated voltages on the thorax model with homogeneous *σ*_BG_ and the temporal average of measured raw EIT voltages during baseline (defined as the last minute of task T2). The difference EIT images were then reconstructed with respect to the baseline and *σ*_BG_ was added to each voxel. In this way, an approximative but simple absolute EIT reconstruction was performed.

### Data analysis

**Fig 4:**
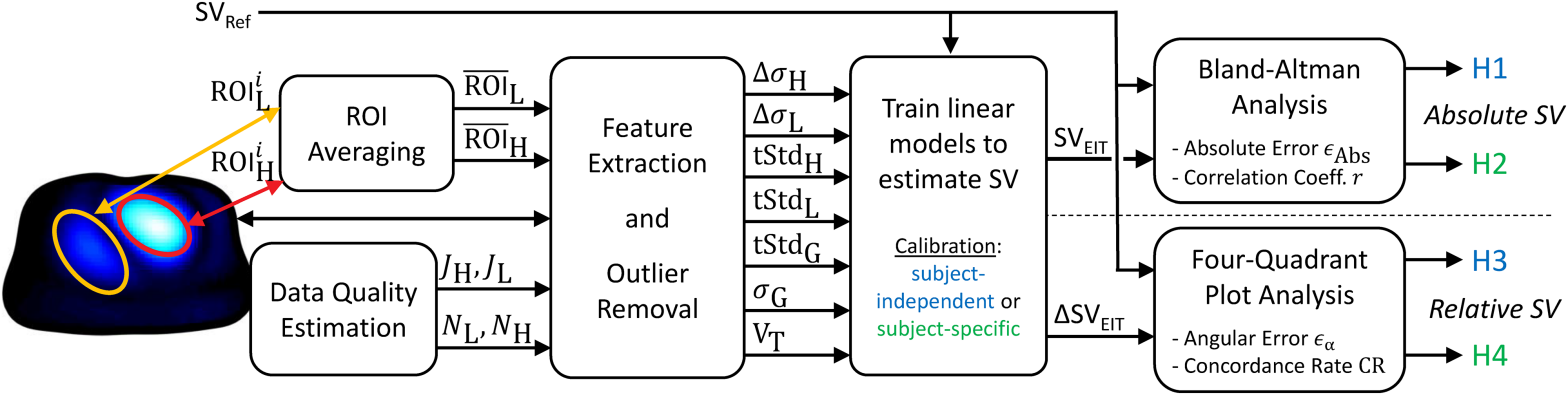
Block diagram of the data analysis. To test the four hypotheses (H1 to H4), different features were extracted from the EIT images and potential outliers with high noise or unstable heart and lung ROIs were removed. Then, the ability to estimate SV with these features via a linear model was evaluated by means of Bland-Altman analysis (absolute SV_EIT_ in H1 and H2) or four-quadrant plot analysis (relative ΔSV_EIT_ in H3 and H4).

In the present study we tested four hypotheses (H1 to H4), namely whether EIT can be used to either estimate absolute SV (H1 and H2) or to trend relative changes of SV (H3 and H4). For H1 and H3 we used a subject-independent and for H2 and H4 a subject-specific calibration. This analysis is detailed in the current section and illustrated and briefly described in Fig 4.

First, from each EIT image sequence of the one-minute averages, the heart and lung regions were determined using the following algorithms: the heart was detected as described in [33, 34] and the lungs via the algorithm proposed by Proença et al. [35, 36]. For each subject an average heart and lung region was calculated and used for the subsequent calculations. To this end, the current ROI (${\text{ROI}}_{H}^{i}$ or ${\text{ROI}}_{L}^{i}$) of the measurement *i* was averaged to the per-subject average (${\bar{\text{ROI}}}_{H}$ or ${\bar{\text{ROI}}}_{L}$), i.e. ${\bar{\text{ROI}}}_{H}$ or ${\bar{\text{ROI}}}_{L}$ contain the biggest connected regions of heart or lung voxels which are present in at least 50% (determined heuristically) of the individual ROIs.

Second, a variety of features were extracted from the EIT images: 1. Δ*σ*_H_, the systolic heart amplitude as the difference of maximum vs minimum in the temporal signal of the heart region; 2. Δ*σ*_L_, the systolic lung amplitude, same as Δ*σ*_H_ but for the lung region; 3. tStd_H_, the heart amplitude as the standard deviation (STD) of the temporal signal in the heart region; 4. tStd_L_, the lung amplitude, same as tStd_H_ but for the lung region; 5. tStd_G_, the global amplitude as the STD of the temporal signal of the sum over all voxels; 6. *σ*_G_, the global conductivity as the mean absolute value of all voxels; 7. V_T_, the average tidal volume as the peak-to-peak respiratory amplitude from the sum signal over all voxels. The latter two were calculated prior to ensemble averaging and high-pass filtering.

Then, assuming a linear relationship between changes in SV and these features, various linear models were trained and evaluated to test the following four hypotheses:

(H1) **Absolute SV with subject-independent calibration**: For each subject a linear model was trained using all other subjects via leave-one-out cross-validation. The resulting performance was evaluated by means of absolute error *ϵ*_Abs_ (in mL) between SV_EIT_ and SV_Ref_ resulting from Bland-Altman analysis [37] and correlation coefficient *r* between SV_EIT_ and SV_Ref_. Measurements were considered as acceptable if *r* ≥ 0.7 (educated guess) and the 95% limits of agreement of *ϵ*_Abs_ did not exceed ±24 mL (= ±30%—the error reported for thermodilution measurements [38]—of the average SV_Ref_).
(H2) **Absolute SV with subject-specific calibration**: This is identical to the first hypothesis (H1), except that a linear model was trained for each subject individually.
(H3) **Trending of relative SV with subject-independent calibration**: For this and the next hypothesis, the features as well as the reference SV were set relative to an initial baseline (The baseline state was defined as the average of the measurements having the three—educated guess—lowest values of SV_Ref_). This leads to the measurement of changes ΔSV_EIT_ which are compared to changes in the reference ΔSV_Ref_ by means of four-quadrant plot analysis [38, 39]. To this end, we quantify the trending ability by means of (1) the angular error *ϵ*_*α*_ and (2) the angular concordance rate CR. (1) *ϵ*_*α*_ is defined as the angle between the identity line (ΔSV_EIT_ = ΔSV_Ref_) and the line from the origin to the point (ΔSV_Ref_, ΔSV_EIT_); (2) CR represents the percentage of measurements with an angular error within a given threshold of *ϵ*_*α*_ ≤ ±30%, which is more restrictive than the traditional concordance rate (including all measurements lying in the 1st and 3rd quadrant). For each subject a linear model was trained using all other subjects via leave-one-out cross-validation. Measurements with CR ≥ 92%, a bias of *ϵ*_*α*_ ≤ ± 5° and its 95% limits of agreement ≤ ± 30°, were considered as acceptable, according to the thresholds suggested by Critchley et al. [40]. The exclusion zone of the four-quadrant plot was set to ±30°—the error for thermodilution measurements [38].
(H4) **Trending of relative SV with subject-specific calibration**: This is identical to the third hypothesis (H3), except that a linear model was trained for each subject individually.

Finally, to limit the analysis to reliable data, four data quality measures were introduced: (1) a similarity measure *J*_*H*_ for the heart region of interest (ROI) comparing the current ${\text{ROI}}_{H}^{i}$ of the measurement *i* to the per-subject average ${\bar{\text{ROI}}}_{H}$ via the so-called *Jaccard index* ($J_{H}=|{\text{ROI}}_{H}^{i}\cap {\bar{\text{ROI}}}_{H}|/|{\text{ROI}}_{H}^{i}\cup {\bar{\text{ROI}}}_{H}|$) [41]; (2) the same similarity measure as *J*_*H*_ but for the lung ROI denoted as *J*_*L*_; (3) *N*_*H*_ and (4) *N*_*L*_ as signal quality indicators estimating the average noise level in the heart and lung region from the relative deviation of each pulse used for ensemble averaging. More details concerning these quality measures can be found in [34]. Only measurements with *J*_*H*_ ≥ 75%, *J*_*L*_ ≥ 75%, *N*_*H*_ > 2.0 and *N*_*L*_ > 2.0 were considered for analysis. The threshold of *N*_*L*_ and *N*_*H*_ were determined based on visual analysis of ensemble averaged signals.

Moreover, the raw EIT data of subject S07 showed severe issues with electrode contact impedance leading to corrupted EIT images. It was therefore completely removed from analysis.

## Results and discussion

### General overview of EIT data

**Fig 5:**
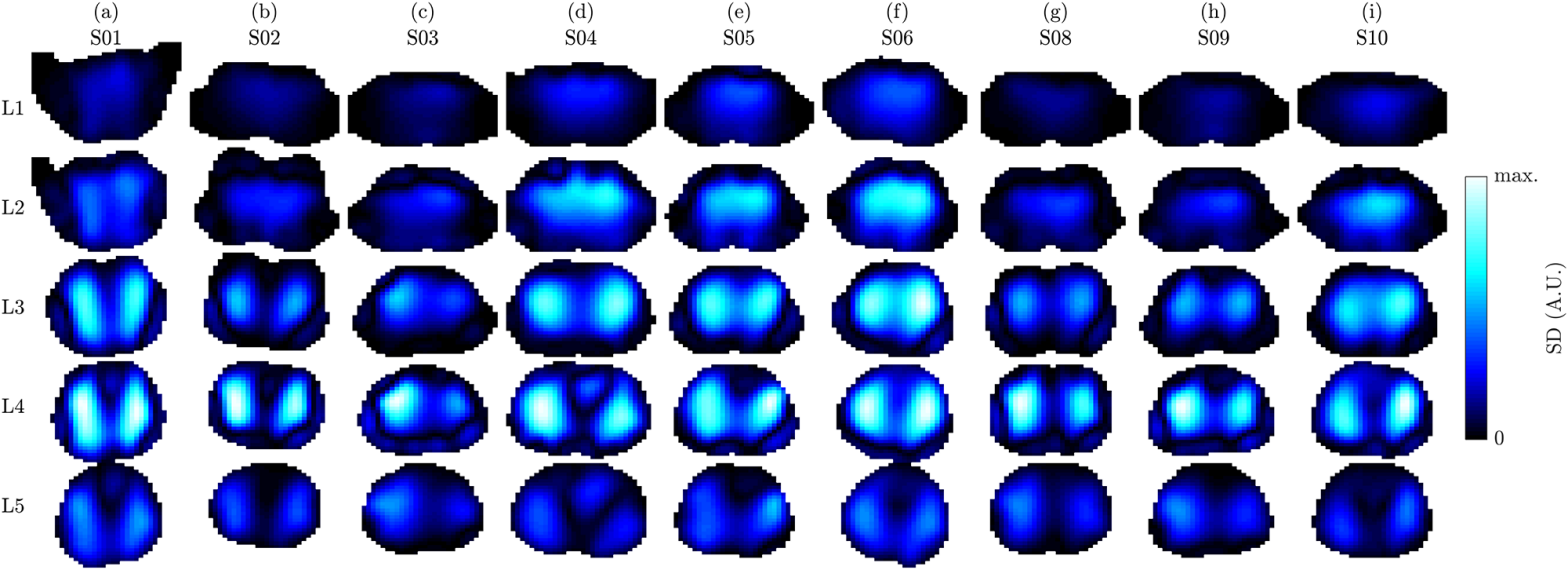
3D EIT images showing the respiration by means of standard deviation (SD) images on the five planes L1 (highest) to L5 (lowest) for the nine volunteers (a) to (i), in supine position. The images of each subject (each column) were scaled to an individual color scale and show the last minute in the first recovery sequence (task T5). Prior to SD calculation the images were filtered using a 2nd-order Butterworth bandpass with *f*_*c*_ = {0.04, 0.5} Hz.

Fig 5 exemplifies respiratory activity of each volunteer by means of standard deviation (SD) images. The strongest respiratory activity can be observed at the lower electrode plane (L4) or in between the two planes (L3). Moreover, the two lung lobes appear separated in the lower image planes (L3 to L5) and more unified in the upper planes (L1 to L2), which is comparable to the observations by Karsten et al. [42].

**Fig 6:**
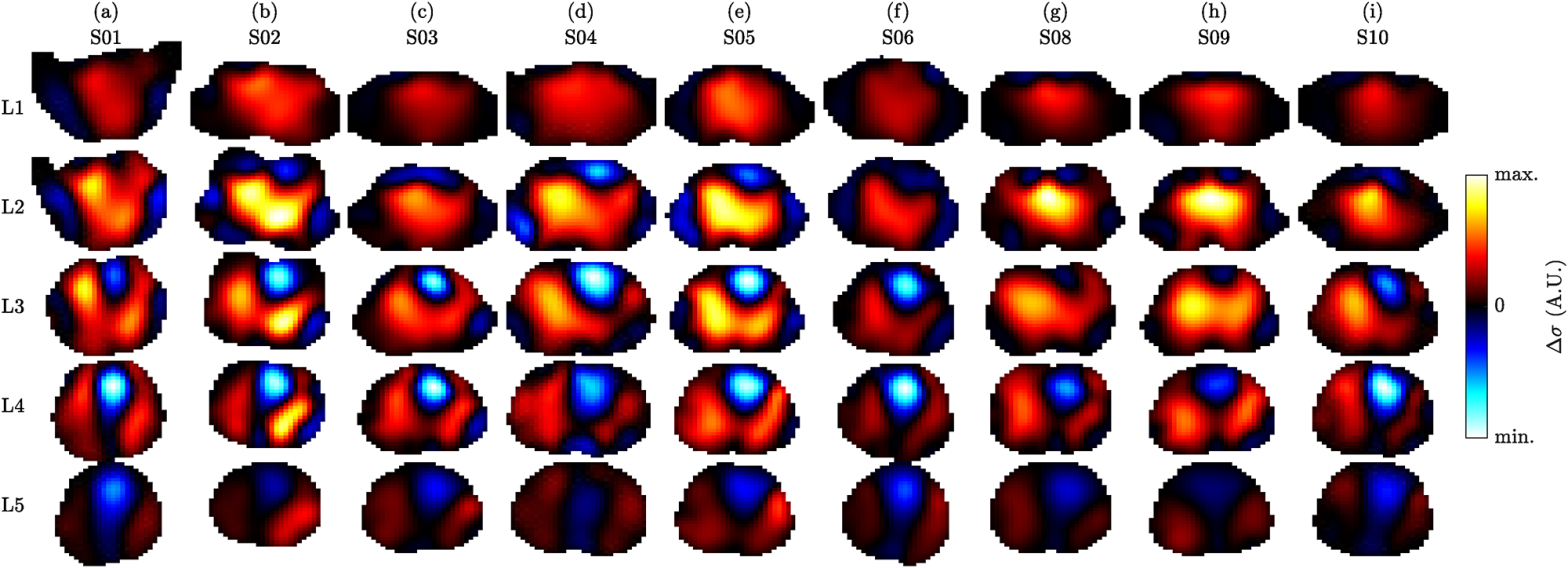
ECG-gated 3D EIT images showing the conductivity difference (end systole vs end diastole) on the five planes L1 (highest) to L5 (lowest) for the nine volunteers (a) to (i), in supine position. The images of each subject (each column) were scaled to an individual color scale and show the average of the last minute in the first recovery sequence (task T5).

Fig 6 shows ECG-gated EIT images by the example of one measurement (last minute of the first recovery sequence—task T5) for the nine subjects analyzed. One can observe that the potential heart regions (blue-white with Δ*σ* < 0) are located in the middle (L3) or lower image plane (L4). On the contrary, the potential lung regions (red-yellow with Δ*σ* > 0) are more present in the upper (L2) or middle image plane (L3). This is in line with the anatomy (i.e. the large pulmonary arteries are located more cranial when compared to the heart, which itself is lower, sitting right on the diaphragm) and observations by Smit et al. [43] who use a high belt placement for cardiovascular EIT of the lungs. Besides, when compared to the other subjects, S08 and S09 show only little activity in the heart with respect to the lung region. It is assumed that for these subjects the lower electrodes were placed too high which decreases the sensitivity in the heart region. A video of the same data is available in S1 Video.

**Fig 7:**
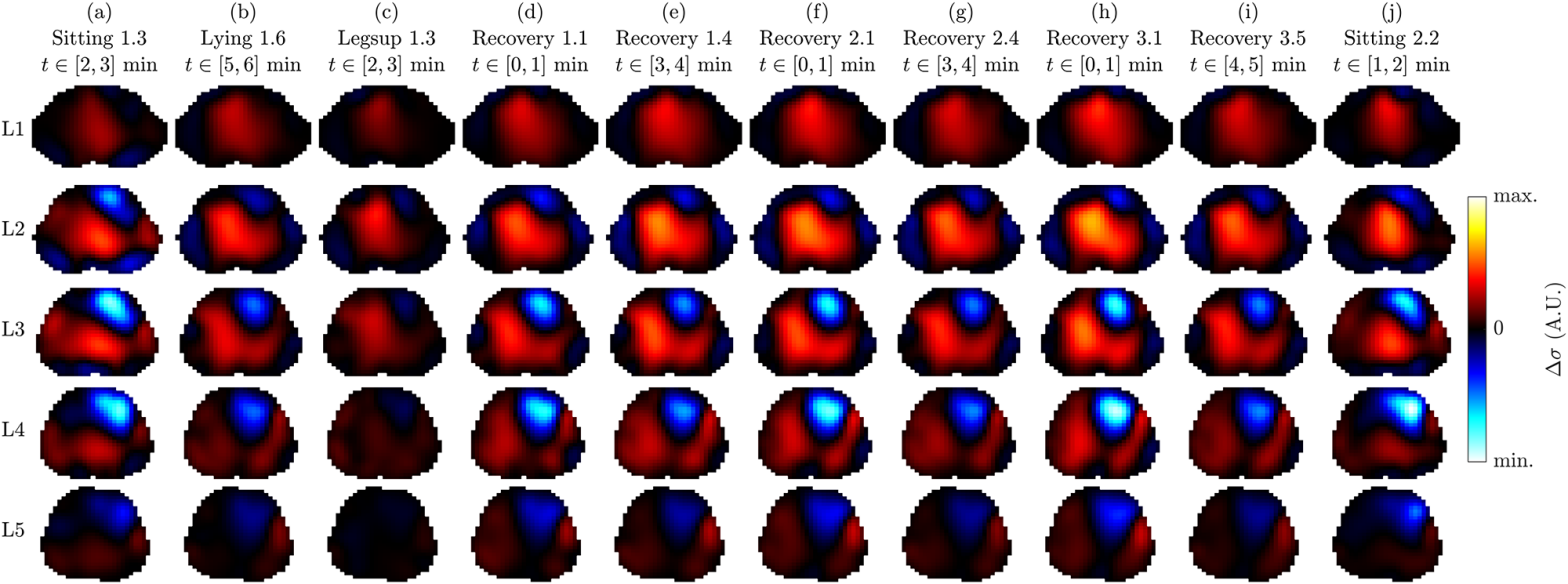
ECG-gated 3D EIT images showing the conductivity difference (end systole vs end diastole) on the five planes L1 (highest) to L5 (lowest) for a selection of ten measurements (a) to (j) of volunteer S05. All images are shown in a common color scale. Note that the last line in the title below each task name indicates the time range (relative to the start of the corresponding task) over which the one-minute average was performed to obtain one representative cardiac cycle.

The ECG-gated EIT images shown in Fig 7 represent different tasks of the same subject. One can observe a significant difference in spatial conductivity distribution between the following three groups of recordings: (1) sitting in (a) and (j), (2) lying with legs up in (c), and (3) the remaining recordings in supine position. These differences were observed for all subjects and are hypothesized to stem from posture-induced heart and lung displacement as well as gravity-induced liquid redistribution in the lungs. On the other hand, when limiting the analysis to the third group of recordings (i.e. all tasks in supine position, except for lying with legs up), the spatial conductivity distribution remains comparable while mainly the amplitude changes.

The high variability observed between these three groups could lead to changes in the ROIs and also their amplitudes which are not necessarily related to changes in SV. Therefore, to limit our analysis to more controlled scenarios and to make it comparable with previous studies [9, 10, 12] (all measured in supine position), only measurements of the last group were considered, i.e. those recorded in supine position (T2, T5, T6, T9, T11 and T13). From the nine subjects remaining for analysis (S07 was excluded as mentioned before), a total of 242 one-minute sequences were available. From these, 11 (4.5%) and 4 (1.7%) were excluded because of a too high noise level in the heart (*N*_*H*_ > 2.0) and lungs (*N*_*L*_ > 2.0), respectively. Then, 76 (31.4%) and 0 were excluded due to too high variability of the heart (*J*_*H*_ < 75%) and lung region (*J*_*L*_ < 75%), as specified in the methods section. The remaining 151 (62.4%) one-minute sequences represent controlled measurements (low noise, stable heart and lung regions, all acquired in supine position), which were further used to investigate the feasibility of EIT-based SV monitoring as presented in the next four sections.

### Hypothesis 1: Absolute SV with subject-independent calibration

In the current and the following section we report on the feasibility of EIT to determine absolute values of SV (in mL).

Row (H1) in Table 1 shows the overall performance (in terms of absolute error *ϵ*_Abs_ and correlation coefficient *r*) for a selection of features tested when using a subject-independent (leave-one-out) calibration. One can observe that for none of the eight features an acceptable performance can be achieved. This confirms our previous observations and the findings by other researchers [10, 12] that a subject-specific calibration is required for absolute SV estimation.

**Table 1:** Overall performance for a selection of features and the four hypotheses. (H1) absolute SV via subject-independent calibration, (H2) absolute SV via subject-specific calibration, (H3) relative SV via subject-independent calibration, and (H4) relative SV via subject-specific calibration. (H1) and (H2) are evaluated in terms of absolute error *ϵ*_Abs_ and correlation coefficient *r* between SV_EIT_ and SV_Ref_. (H3) and (H4) are evaluated in terms of angular error *ϵ*_*α*_ and angular concordance rate CR between ΔSV_EIT_ and ΔSV_Ref_. The (†) indicates unrealistic solutions with calibrations coefficients *not* having identical sign for all subjects. Cell shadings indicate whether the acceptance criteria (see methods section) are met (green), not met (red), or met but with unrealistic calibration coefficients (yellow). The errors *ϵ*_Abs_ and *ϵ*_*α*_ are given as Mean ± Std and the 95% limits of agreement correspond to [Mean − 1.96 ⋅ Std, Mean + 1.96 ⋅ Std].

	Absolute SV				Trending of Relative SV			
	(H1) Hypothesis 1		(H2) Hypothesis 2		(H3) Hypothesis 3		(H4) Hypothesis 4	
	*ϵ*_Abs_ (mL)	*r* (1)	*ϵ*_Abs_ (mL)	*r* (1)	*ϵ*_*α*_ (°)	CR (%)	*ϵ*_*α*_ (°)	CR (%)
Δ*σ*_H_	−0.5 ± 28.2	−0.424	0.0 ± 15.2	0.813	−5.3 ± 25.2	76.9	−1.0 ± 23.0	80.9
tStd_H_	−1.0 ± 27.3	0.023	(†) 0.0 ± 14.3	0.836	−4.9 ± 26.5	73.8	(†) −3.9 ± 21.5	83.3
Δ*σ*_L_	−0.4 ± 27.3	−0.023	(†) 0.0 ± 15.8	0.796	−12.1 ± 20.3	70.4	(†) −0.2 ± 22.5	84.6
tStd_L_	−0.5 ± 28.1	−0.341	(†) 0.0 ± 17.1	0.755	−17.4 ± 16.7	70.2	(†) −5.8 ± 20.4	91.5
tStd_G_	(†) −0.5 ± 28.2	−0.710	(†) 0.0 ± 16.8	0.766	(†) −15.6 ± 25.7	73.3	(†) 2.0 ± 24.2	74.4
Δ*σ*_H_, Δ*σ*_H_/*σ*_G_	−1.7 ± 30.4	−0.365	0.0 ± 10.4	0.917	−1.9 ± 20.4	83.9	1.0 ± 17.5	87.7
Δ*σ*_L_, Δ*σ*_L_/*σ*_G_	(†) −0.4 ± 28.4	−0.050	0.0 ± 10.3	0.920	−1.7 ± 21.7	84.2	1.3 ± 16.7	93.0
V_T_	−0.4 ± 24.7	0.371	0.0 ± 9.7	0.929	−1.5 ± 18.5	89.8	−0.4 ± 15.3	94.7

Subject-specific performances for hypothesis (H1) are given in the appendix in S1 Table.

### Hypothesis 2: Absolute SV with subject-specific calibration

**Fig 8:**
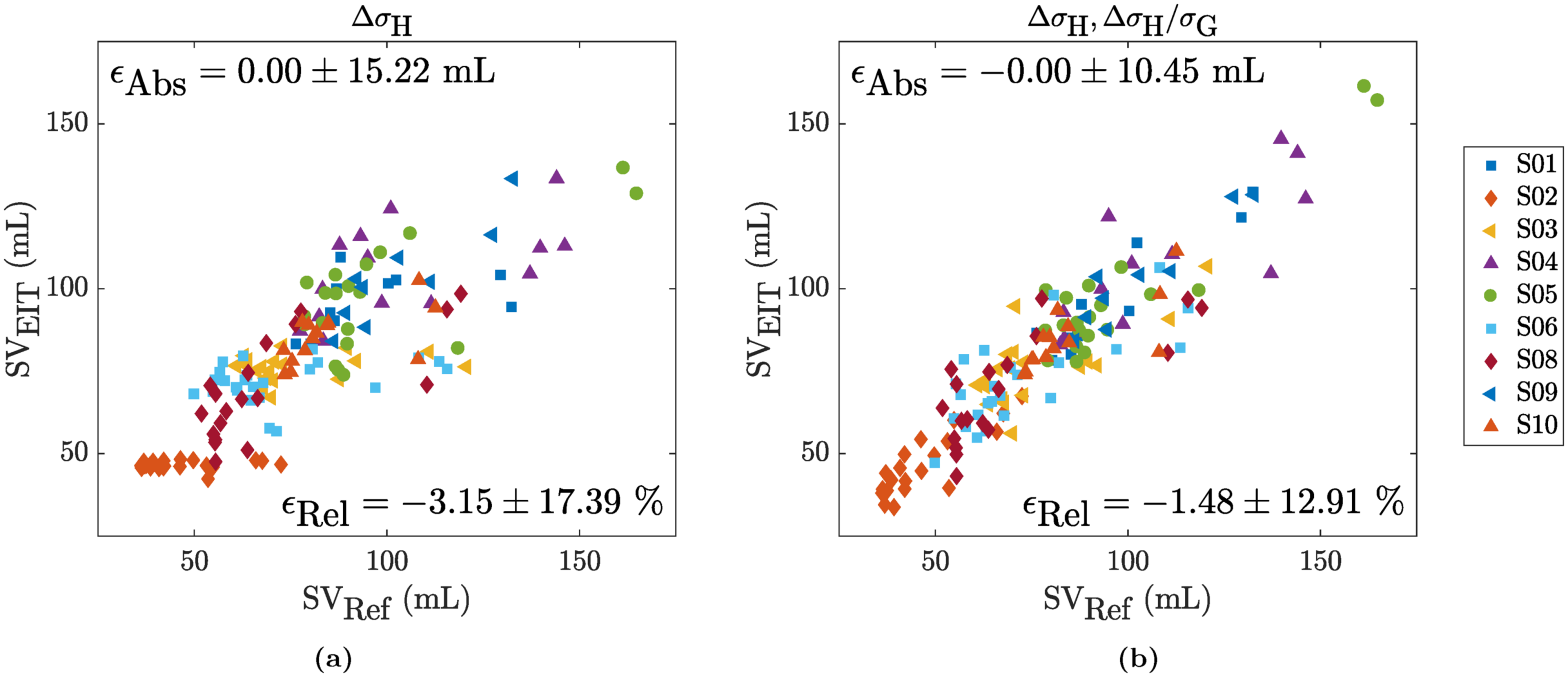
SV_EIT_ vs SV_Ref_ for a subject-specific calibration in hypothesis (H2) with the features (a) Δ*σ*_H_ or (b) Δ*σ*_H_ and $\frac{\Delta \sigma_{H}}{\sigma_{G}}$.

**Fig 9:**
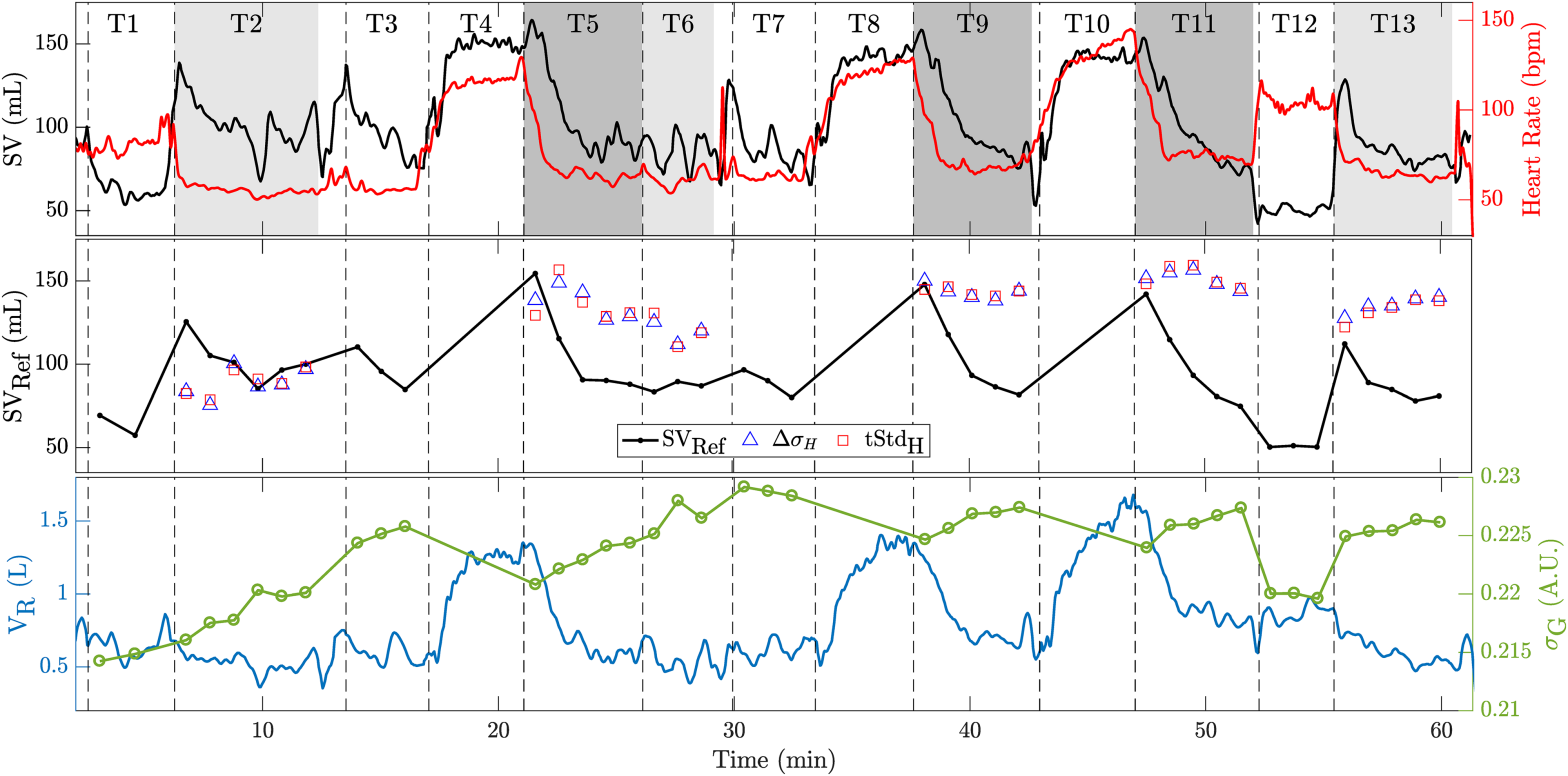
(Top) Example of temporal evolution of reference stroke volume (black) and heart rate (red) for the entire protocol comprised of tasks T1 to T13 (see Fig 1) for subject S03. The beginning of each task is marked with a dashed vertical line and the particular tasks considered for analysis are shaded in light (lying) or dark gray (recovery). (Middle) One-minute averages used for analysis showing SV_Ref_ and two EIT features: the systolic heart amplitude (Δ*σ*_H_) and the temporal standard-deviation of the heart signal during one full cardiac cycle (tStd_H_). (Bottom) Tidal volume V_R_ (blue) measured by the reference device (MetaMax 3B) and the one-minute averages of the global conductivity feature *σ*_G_ (green).

Row (H2) in Table 1 shows the overall performance when using a subject-specific calibration. When concentrating the analysis on the five amplitude features (Δ*σ*_H_, tStd_H_, Δ*σ*_L_, tStd_L_, tStd_G_), one can observe that all of the overall errors have limits of agreement exceeding the ±24 mL threshold (= ±30% of the average SV_Ref_ as specified in the methods section). Moreover, except for Δ*σ*_H_, no uniform calibration could be found with either all positive or negative calibration coefficients (marked with a (†)). The relationship between SV_EIT_ and SV_Ref_ of the feature Δ*σ*_H_ is shown in Fig 8a. One can observe that at least for subject S03, SV_EIT_ does not at all follow the changes in SV_Ref_. This particular case of S03 is illustrated in more detail in Fig 9 (Middle) by means of the temporal evolution of SV_Ref_ in comparison to the two features related to the heart amplitude (tStd_H_ and Δ*σ*_H_). The same figures for the remaining subjects are available as supporting information in S1 to S8 Figs. It is obvious from these findings that—for the present data—changes in the heart-related amplitude (tStd_H_ or Δ*σ*_H_) are not solely related to changes in SV. This is in line with the findings from simulations [33], that the EIT heart signal is influenced by other factors and—among others—scaled with the heart-lung-conductivity contrast (difference of heart vs lung conductivity).

When taking into account the global conductivity *σ*_G_ to normalize the systolic heart amplitude Δ*σ*_H_ (i.e. SV_EIT_ = *κ*_0_ + *κ*_1_ ⋅ Δ*σ*_H_ + *κ*_2_ ⋅ Δ*σ*_H_/*σ*_G_) the absolute error can be reduced to ±10.45 mL as shown in Fig 8b and listed in Table 1(H2). It is known from simulations [33] that the EIT heart amplitude is scaled by the aforementioned heart-lung-conductivity contrast (HLC). As *σ*_G_ contains information about the lung conductivity, it is hypothesized that it serves as a rough estimate of the HLC and thus allows for correction of this scaling. While the exact physiological background is not fully understood, it still shows that normalizing Δ*σ*_H_ by *σ*_G_ can lead to improved results. A similar reduction in error can be achieved when normalizing the lung amplitude by *σ*_G_. A possible reason might be that the lung amplitude estimates are similarly affected by changes in global conductivity and thus require normalization.

However, it needs to be mentioned that in the current protocol the EIT-derived tidal volume V_T_ is highly correlated with changes in SV (average corr. coefficient $\bar{r}=0.85$, range *r* ∈ [0.59, 0.96]) as also shown by the low absolute error for V_T_ in Table 1(H2). At the same time the global conductivity *σ*_G_ is influenced by the tidal volume V_T_ (V_T_ ↑ ⇒ *σ*_G_ ↓). Nonetheless, *σ*_G_ has other influencing factors such as the content of liquid in the lungs (e.g. blood or water), the posture (including the position of the torso and the arms [44]) and the contact impedance of EIT electrodes (i.e. varying external pressure on electrodes can lead to changes in global conductivity [45]). Based on the current protocol, it can neither be excluded nor fully confirmed that using the normalized heart (Δ*σ*_H_/*σ*_G_) or lung amplitudes (Δ*σ*_L_/*σ*_G_) leads to an improved estimation of SV (as in this protocol the latter is highly correlated to V_T_ which in turn is related to 1/*σ*_G_).

Subject-specific performances for hypothesis (H2) are given in the appendix in S2 Table.

### Hypothesis 3: Relative SV with subject-independent calibration

In the current and the following section we report on the feasibility of EIT to perform trending of SV, that is following the relative change ΔSV_EIT_ (in %) with respect to an initial baseline value.

**Fig 10:**
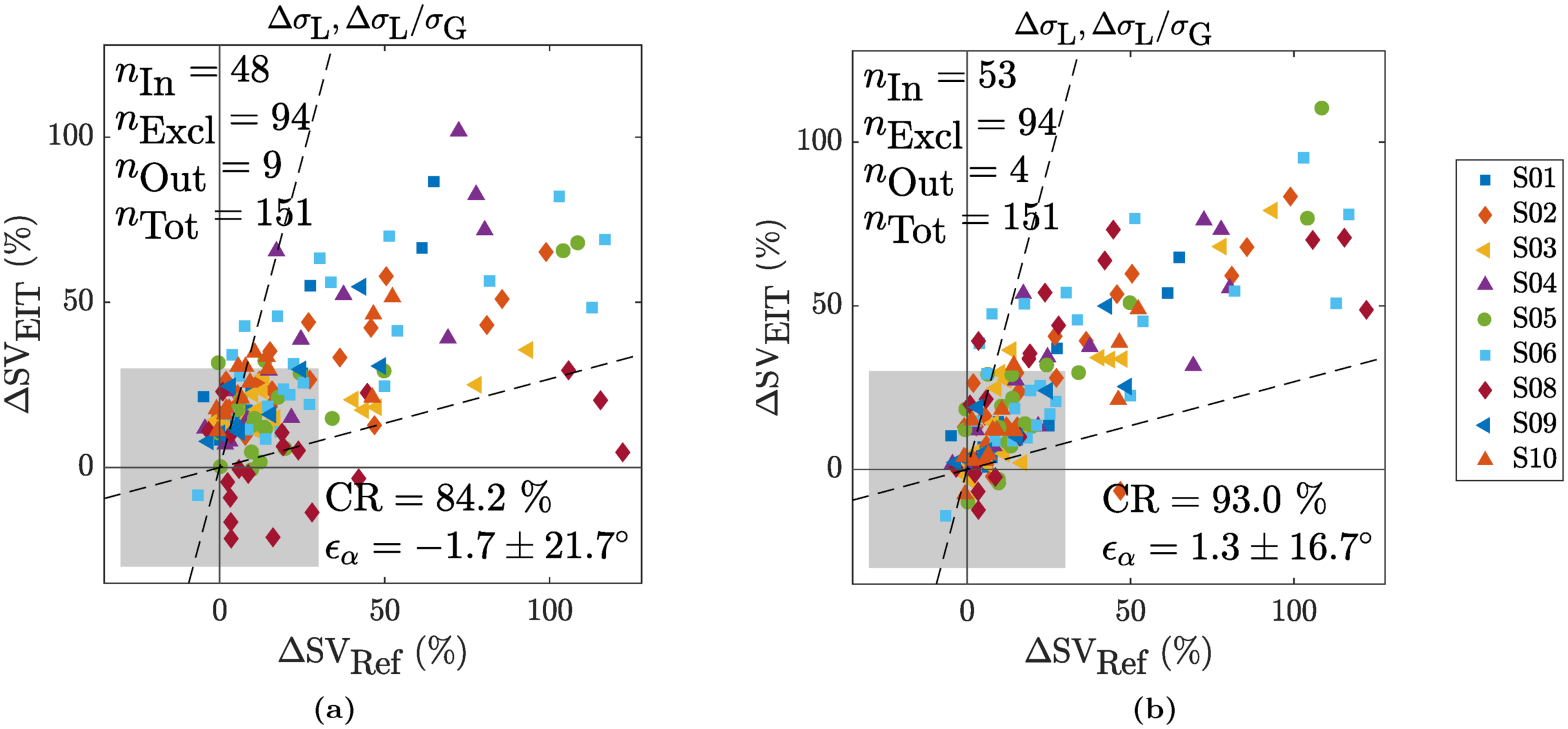
Trending ability of ΔSV_EIT_ vs ΔSV_Ref_ shown by means of four-quadrant plots for the combination of the two features Δ*σ*_L_ and $\frac{\Delta \sigma_{L}}{\sigma_{G}}$ and (a) a subject-independent calibration for hypothesis (H3) or (b) a subject-specific calibration for hypothesis (H4). The exclusion zone was set to ±30%.

The performances obtained for a subject-independent (leave-one-out) calibration are listed in Table 1(H3). For none of the features tested, an acceptable trending performance can be obtained. An example is given in Fig 10a for the normalized lung amplitude (Δ*σ*_L_, Δ*σ*_L_/*σ*_G_) which leads to the best performance in terms of CR (when not considering V_T_).

Subject-specific performances for hypothesis (H3) are given in the appendix in S3 Table.

### Hypothesis 4: Relative SV with subject-specific calibration

The performances obtained when using a subject-specific calibration are listed in Table 1(H4). While the four simple features (tStd_H_, Δ*σ*_L_, tStd_L_ and tStd_G_) do not lead to realistic calibration factors with a same sign, Δ*σ*_H_ does. However, none of these features fulfill the trending requirements in terms of *ϵ*_*α*_ and CR. This also applies to the normalized versions of (Δ*σ*_H_, Δ*σ*_H_/*σ*_G_) and (Δ*σ*_L_, Δ*σ*_L_/*σ*_G_). Even though they have a CR very close to or above the acceptable 92%, they both exceed the acceptance threshold in terms of 95% limits of agreement with [-33.3, 35.3]° and [-31.4, 34.0]°, respectively. The latter is also shown in Fig 10b.

Subject-specific performances for hypothesis (H4) are given in the appendix in S4 Table.

### Limitations and future work

The present study is limited in that it was performed on healthy volunteers which restricts the reference SV to be measured with noninvasive devices. The SV reference measurement device used is not considered as gold standard [38], as simply no noninvasive gold standard exists. Nonetheless, it is possibly among the most accurate when requiring continuous and noninvasive measurements on healthy subjects performing physical exercise. Besides, in the current experimental protocol, the tidal volume V_T_ is highly correlated to SV (mainly during post-exercise recovery). In addition, the reference method used to estimate SV relies on the measurement of the oxygen uptake ${\dot{V}}_{O_{2}}$ which in turn is related to V_T_. Therefore it is unclear whether this does not even exacerbate the high correlation between V_T_ and SV. To either confirm or reject our hypothesis that *σ*_G_ is useful for normalizing Δ*σ*_H_ and Δ*σ*_L_ —and not simply because it is related to V_T_ —a different experiment protocol should be designed, where SV is less correlated to V_T_.

The subject-specific EIT reconstruction model used was acquired in sitting position while EIT images were mainly analyzed in supine position. In addition, big differences in the spatial conductivity distribution were observed in EIT images between sitting and supine. To exclude the potential influence of the reconstruction model on these differences, an additional model in supine position (e.g. via MRI scans) should be created. Besides, when aiming for EIT-based SV in different body positions, a deeper understanding of the observed differences is crucial.

One could further criticize the manual synchronization performed between EIT and ECG. However, its accuracy was first tested in the laboratory and the error has shown to be below two EIT frames (< ± 40 ms). Moreover, all ensemble averaged sequences were visually verified for physiological meaningful time delays. Even though this approach is sufficient for amplitude-based measures used in the present study, it is not accurate enough for EIT-based timing measures [35, 46] which necessitates an EIT system synchronously measuring ECG [47].

Another limitation of the present study is that it is heavily biased in gender (9 males vs 1 female). Even though no substantial difference in accuracy could be observed for the one female volunteer (S02) when compared to the male volunteers (see S1 to S4 Tables), this needs to be investigated in a larger and more heterogeneous (in terms of gender) population. Future studies should analyze potential differences in performance of EIT-derived SV related to gender as it was observed in previous studies for tidal ventilation [48].

## Conclusion

In this work, we investigated the EIT-based estimation of SV in healthy subjects and compared it to reference measurements derived from the oxygen uptake ${\dot{V}}_{O_{2}}$. Large variations in SV were induced via postural changes and recovery after supine cycling exercise. To minimize known influences of heart and belt displacement on EIT-based SV, 3D EIT with self-adhesive gel electrodes in combination with a subject-specific reconstruction model was applied.

The ECG-gated 3D EIT images show large differences in spatial conductivity distribution between sitting, lying with legs up and supine position. To limit the analysis to very isolated and constant settings, only measurements in supine position were considered and 38.4% of the remaining measurements were excluded due to high noise or unstable heart or lung regions. The temporal amplitudes in the heart (Δ*σ*_H_ and tStd_H_) [9, 10], the lungs (Δ*σ*_L_ and tStd_L_) [11, 12], or in the entire image (tStd_G_) were calculated but none of them showed an accurate relation to the reference SV_Ref_. Therefore, we cannot confirm the recent observations made in pig experiments [10, 11, 12], despite having used a subject-specific 3D EIT measurement setup to minimize effects of electrode displacement or out-of-EIT-plane movement of the heart.

Based on findings from simulations [33], the heart amplitude Δ*σ*_H_ normalized by the global conductivity *σ*_G_ was included as a feature. The resulting linear combination (${\text{SV}}_{\text{EIT}}=\kappa_{0}+\kappa_{1}\cdot \Delta \sigma_{H}+\kappa_{2}\cdot \frac{\Delta \sigma_{H}}{\sigma_{G}}$) leads to more promising results. That is an overall error of 0.0 ± 10.4 mL for absolute SV with a subject-specific calibration. When aiming for the trending of relative changes in SV with the same type of calibration, we achieve a performance of *ϵ*_*α*_ = 1.0 ± 17.5° and CR = 87.7%. Similar results were obtained when using the lung amplitude normalized by *σ*_G_, i.e. ${\text{SV}}_{\text{EIT}}=\kappa_{0}+\kappa_{1}\cdot \Delta \sigma_{L}+\kappa_{2}\cdot \frac{\Delta \sigma_{L}}{\sigma_{G}}$. In contrast, both absolute and relative SV do not seem to be feasible when using a subject-independent calibration.

However, in the current protocol, SV is highly correlated to the tidal volume V_T_, which in turn is related to *σ*_G_. The current findings should therefore be considered with caution since the normalization attempts suggested might primarily lead to a satisfactory outcome because of the relation between V_T_ and *σ*_G_. To either confirm or reject our hypothesis that Δ*σ*_H_ or Δ*σ*_L_ normalized by *σ*_G_ lead to reliable SV estimates, a different experiment protocol is required, where SV is less correlated to V_T_.

In conclusion, we could show that even with a subject-specific 3D EIT setup on healthy volunteers, purely amplitude-based features are very unlikely to provide feasible SV estimates in experimental conditions as they are influenced by other factors (such as lung and heart conductivity, posture and electrode contact impedance). While the normalization of the heart or lung amplitudes via the global conductivity shows promise on the current data, this approach requires confirmation in different experimental protocols.

All the data of the present study are made publicly available (see S1 File) for further investigations.

## Supporting information

S1 Fig Temporal evolution of SV_Ref_, heart rate and EIT-based features for subject S01. See caption of Fig 9 for details.
(PDF)

S2 Fig Temporal evolution of SV_Ref_, heart rate and EIT-based features for subject S02. See caption of Fig 9 for details.
(PDF)

S3 Fig Temporal evolution of SV_Ref_, heart rate and EIT-based features for subject S04. See caption of Fig 9 for details.
(PDF)

S4 Fig Temporal evolution of SV_Ref_, heart rate and EIT-based features for subject S05. See caption of Fig 9 for details.
(PDF)

S5 Fig Temporal evolution of SV_Ref_, heart rate and EIT-based features for subject S06. See caption of Fig 9 for details.
(PDF)

S6 Fig Temporal evolution of SV_Ref_, heart rate and EIT-based features for subject S08. See caption of Fig 9 for details.
(PDF)

S7 Fig Temporal evolution of SV_Ref_, heart rate and EIT-based features for subject S09. See caption of Fig 9 for details.
(PDF)

S8 Fig Temporal evolution of SV_Ref_, heart rate and EIT-based features for subject S10. See caption of Fig 9 for details.
(PDF)

S1 Table Absolute SV via subject-independent calibration on healthy volunteers. Subject-specific and overall performance for a selection of eight features (a) to (h) and hypothesis (H1) absolute SV via subject-independent calibration. The performance between SV_EIT_ and SV_Ref_ is evaluated in terms of absolute error *ϵ*_Abs_ and correlation coefficient *r*. The (†) indicates unrealistic solutions with calibrations coefficients *not* having identical sign for all subjects. Cell shadings indicate whether the acceptance criteria (see methods section) are met (green), not met (red), or met but with unrealistic calibration coefficients (yellow).
(PDF)

S2 Table Absolute SV via subject-specific calibration on healthy volunteers. Subject-specific and overall performance for a selection of eight features (a) to (h) and hypothesis (H2) absolute SV via subject-specific calibration. The performance between SV_EIT_ and SV_Ref_ is evaluated in terms of absolute error *ϵ*_Abs_ and correlation coefficient *r*. The (†) indicates unrealistic solutions with calibrations coefficients *not* having identical sign for all subjects. Cell shadings indicate whether the acceptance criteria (see methods section) are met (green), not met (red), or met but with unrealistic calibration coefficients (yellow).
(PDF)

S3 Table Relative SV via subject-independent calibration on healthy volunteers. Subject-specific and overall performance for a selection of eight features (a) to (h) and hypothesis (H3) relative SV via subject-independent calibration. The performance between ΔSV_EIT_ and ΔSV_Ref_ is evaluated in terms of angular error *ϵ*_*α*_ and angular concordance rate CR. The (†) indicates unrealistic solutions with calibrations coefficients *not* having identical sign for all subjects. Cell shadings indicate whether the acceptance criteria (see methods section) are met (green), not met (red), or met but with unrealistic calibration coefficients (yellow).
(PDF)

S4 Table Relative SV via subject-specific calibration on healthy volunteers. Subject-specific and overall performance for a selection of eight features (a) to (h) and hypothesis (H4) relative SV via subject-specific calibration. The performance between ΔSV_EIT_ and ΔSV_Ref_ is evaluated in terms of angular error *ϵ*_*α*_ and angular concordance rate CR. The (†) indicates unrealistic solutions with calibrations coefficients *not* having identical sign for all subjects. Cell shadings indicate whether the acceptance criteria (see methods section) are met (green), not met (red), or met but with unrealistic calibration coefficients (yellow).
(PDF)

S1 Video Video of ECG-gated 3D EIT for each subject. Sequence of images showing the cardiosynchronous conductivity change on the five image planes L1 (highest) to L5 (lowest) for the nine volunteers (a) to (i), in supine position. For each subject (i.e. each column) the images were scaled to an individual color scale and show the average of the last minute in the first recovery sequence (task T5). The cardiac cycle of each subject was normalized to 60 bpm. The video speed is half the real speed. The difference images shown are relative to the minimum of each voxel.
(AVI)

S1 File Data and scripts. (TXT)
