# Supplementary material for: Accuracy and reliability of noninvasive stroke volume monitoring via ECG-gated 3D electrical impedance tomography in healthy volunteers
Source: PLoS One. 2018 Jan 26;13(1):e0191870. doi: 10.1371/journal.pone.0191870 (PMC5786320; doi:10.1371/journal.pone.0191870)
Supplement: S6 Fig — See caption of Fig 9 for details. (PDF) [file pone.0191870.s006.pdf]

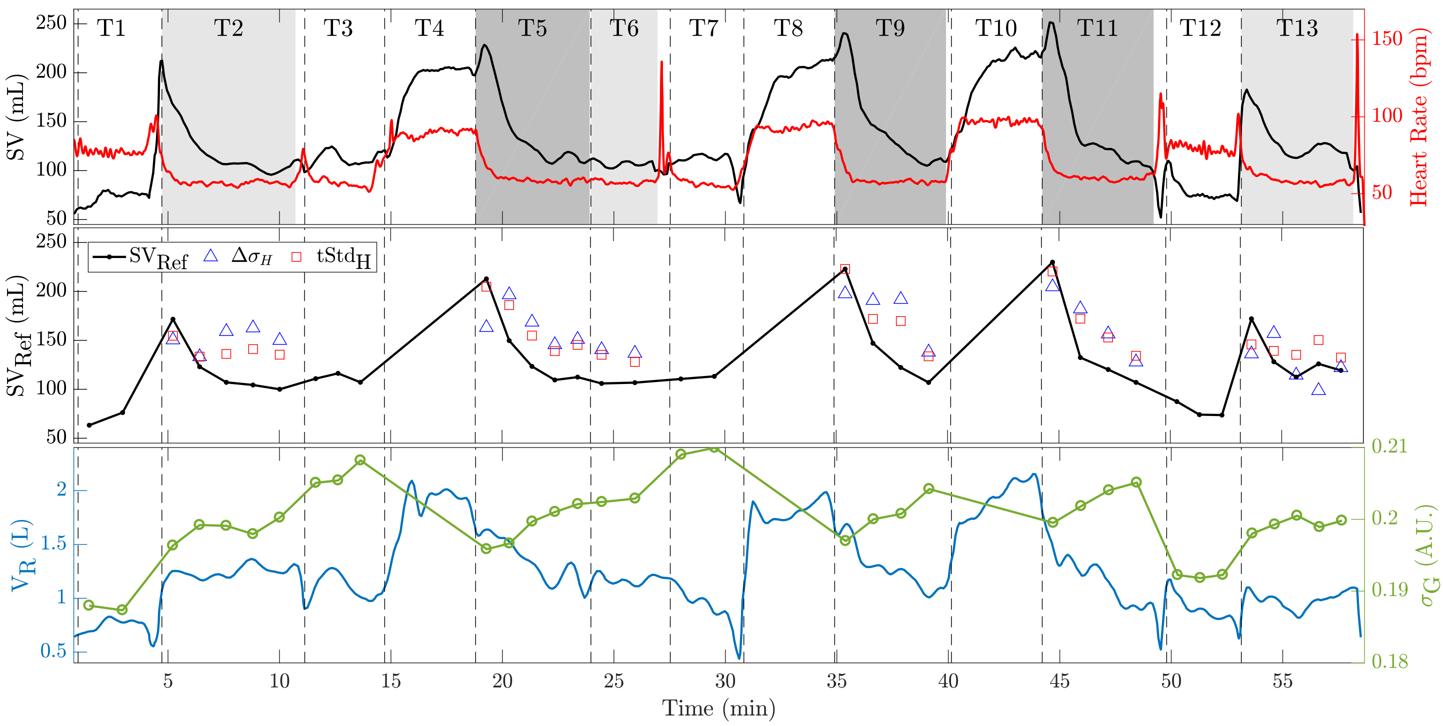


**Fig S6.** Temporal evolution of $SV_{Ref}$, heart rate and EIT-based features for subject S08. See caption of Fig. 9 for details.
