# Supplementary material for: Accuracy and reliability of noninvasive stroke volume monitoring via ECG-gated 3D electrical impedance tomography in healthy volunteers
Source: PLoS One. 2018 Jan 26;13(1):e0191870. doi: 10.1371/journal.pone.0191870 (PMC5786320; doi:10.1371/journal.pone.0191870)
Supplement: S7 Fig — See caption of Fig 9 for details. (PDF) [file pone.0191870.s007.pdf]

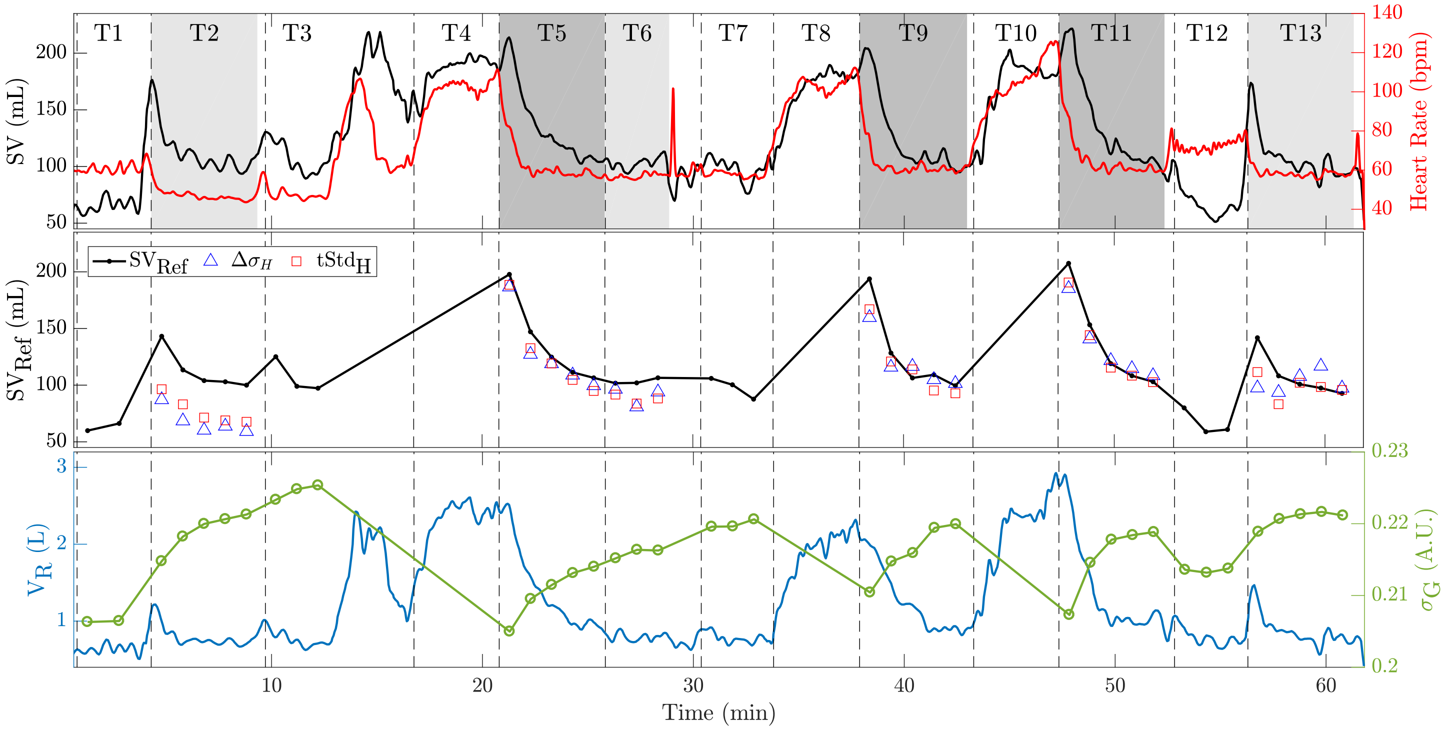

Fig S7. Temporal evolution of SV_{Ref}, heart rate and EIT-based features for subject S09. See caption of Fig. 9 for details.
