# Supplementary material for: Accuracy and reliability of noninvasive stroke volume monitoring via ECG-gated 3D electrical impedance tomography in healthy volunteers
Source: PLoS One. 2018 Jan 26;13(1):e0191870. doi: 10.1371/journal.pone.0191870 (PMC5786320; doi:10.1371/journal.pone.0191870)
Supplement: S1 Table — Subject-specific and overall performance for a selection of eight features (a) to (h) and hypothesis (H1) absolute SV via subject-independent calibration. The performance between SVEIT and SVRef is evaluated in terms of absolute error ϵAbs and correlation coefficient r. The (†) indicates unrealistic solutions with calibrations coefficients not having identical sign for all subjects. Cell shadings indicate whether the acceptance criteria (see methods section) are met (green), not met (red), or met but with unrealistic calibration coefficients (yellow). (PDF) [file pone.0191870.s009.pdf]

Table S1. Subject-specific and overall performance for a selection of eight features (a) to (h) and hypothesis (H1) absolute SV via subject-independent calibration. The performance between SV_{EIT} and SV_{Ref} is evaluated in terms of absolute error ϵ_{Abs} and correlation coefficient r. The (†) indicates unrealistic solutions with calibrations coefficients *not* having identical sign for all subjects. Cell shadings indicate whether the acceptance criteria (see methods section) are met (green), not met (red), or met but with unrealistic calibration coefficients (yellow).

	(a) $\Delta\sigma_H$		(b) $tStd_H$		(c) $\Delta\sigma_L$		(d) $tStd_L$	
	ϵ_{Abs} (mL)	r (1)	ϵ_{Abs} (mL)	r (1)	ϵ_{Abs} (mL)	r (1)	ϵ_{Abs} (mL)	r (1)
S01	18.7 ± 18.0	0.456	11.7 ± 17.8	0.358	21.6 ± 14.8	0.714	17.9 ± 17.9	0.467
S02	−39.5 ± 11.0	0.118	−38.9 ± 12.6	−0.144	−37.2 ± 13.0	−0.070	−39.4 ± 11.9	−0.035
S03	−7.9 ± 16.2	0.242	−12.8 ± 17.0	0.102	−9.1 ± 24.9	−0.786	−5.6 ± 21.3	−0.753
S04	30.5 ± 23.9	0.563	29.2 ± 23.4	0.426	30.6 ± 20.6	0.784	28.2 ± 24.3	0.358
S05	21.0 ± 23.1	0.696	22.9 ± 19.2	0.778	16.6 ± 24.0	0.172	19.9 ± 25.9	−0.167
S06	−10.5 ± 18.1	0.328	−13.4 ± 17.1	0.417	−3.6 ± 18.9	0.152	−7.5 ± 18.3	0.281
S08	−12.0 ± 20.9	0.720	−6.5 ± 15.2	0.958	−11.1 ± 18.8	0.598	−11.2 ± 20.4	0.482
S09	26.4 ± 15.8	0.893	30.8 ± 12.5	0.935	18.5 ± 16.1	0.356	22.0 ± 16.6	0.235
S10	6.3 ± 12.6	0.577	5.7 ± 10.6	0.639	2.1 ± 12.8	0.366	4.1 ± 13.6	0.161
All	−0.5 ± 28.2	−0.424	−1.0 ± 27.3	0.023	−0.4 ± 27.3	−0.023	−0.5 ± 28.1	−0.341

	(e) $tStd_G$		(f) $\Delta\sigma_H, \frac{\Delta\sigma_H}{\sigma_G}$		(g) $\Delta\sigma_L, \frac{\Delta\sigma_L}{\sigma_G}$		(h) V_T	
	ϵ_{Abs} (mL)	r (1)	ϵ_{Abs} (mL)	r (1)	ϵ_{Abs} (mL)	r (1)	ϵ_{Abs} (mL)	r (1)
S01	18.3 ± 18.6	0.363	17.1 ± 15.4	0.884	21.6 ± 12.3	0.833	15.1 ± 5.5	0.963
S02	−38.8 ± 11.2	−0.186	−52.4 ± 6.3	0.833	−39.4 ± 11.6	0.244	−39.8 ± 9.4	0.898
S03	−5.2 ± 18.3	−0.844	−8.3 ± 15.2	0.546	−15.9 ± 27.9	−0.684	−4.4 ± 13.5	0.590
S04	28.5 ± 25.2	0.069	29.9 ± 22.5	0.815	29.9 ± 20.0	0.803	25.9 ± 17.1	0.791
S05	19.3 ± 25.1	−0.600	25.0 ± 21.8	0.755	23.5 ± 19.5	0.870	24.2 ± 12.9	0.954
S06	−10.0 ± 18.9	−0.100	−8.7 ± 17.2	0.676	−4.9 ± 18.0	0.302	−8.6 ± 11.0	0.814
S08	−11.8 ± 21.5	0.093	−11.7 ± 19.2	0.650	−11.5 ± 17.2	0.698	−18.0 ± 11.8	0.860
S09	26.0 ± 16.7	0.649	28.1 ± 15.8	0.844	22.4 ± 12.8	0.768	25.0 ± 10.0	0.907
S10	5.6 ± 13.9	−0.139	5.1 ± 12.3	0.729	6.1 ± 11.8	0.543	11.9 ± 7.1	0.860
All	(†) −0.5 ± 28.2	−0.710	−1.7 ± 30.4	−0.365	(†) −0.4 ± 28.4	−0.050	−0.4 ± 24.7	0.371
