# Supplementary material for: Accuracy and reliability of noninvasive stroke volume monitoring via ECG-gated 3D electrical impedance tomography in healthy volunteers
Source: PLoS One. 2018 Jan 26;13(1):e0191870. doi: 10.1371/journal.pone.0191870 (PMC5786320; doi:10.1371/journal.pone.0191870)
Supplement: S2 Table — Subject-specific and overall performance for a selection of eight features (a) to (h) and hypothesis (H2) absolute SV via subject-specific calibration. The performance between SVEIT and SVRef is evaluated in terms of absolute error ϵAbs and correlation coefficient r. The (†) indicates unrealistic solutions with calibrations coefficients not having identical sign for all subjects. Cell shadings indicate whether the acceptance criteria (see methods section) are met (green), not met (red), or met but with unrealistic calibration coefficients (yellow). (PDF) [file pone.0191870.s010.pdf]

Table S2. Subject-specific and overall performance for a selection of eight features (a) to (h) and hypothesis (H2) absolute SV via subject-specific calibration. The performance between SV_{EIT} and SV_{Ref} is evaluated in terms of absolute error ϵ_{Abs} and correlation coefficient r. The (†) indicates unrealistic solutions with calibrations coefficients *not* having identical sign for all subjects. Cell shadings indicate whether the acceptance criteria (see methods section) are met (green), not met (red), or met but with unrealistic calibration coefficients (yellow).

	(a) $\Delta\sigma_H$		(b) $tStd_H$		(c) $\Delta\sigma_L$		(d) $tStd_L$	
	ϵ_{Abs} (mL)	r (1)	ϵ_{Abs} (mL)	r (1)	ϵ_{Abs} (mL)	r (1)	ϵ_{Abs} (mL)	r (1)
S01	0.0 ±16.9	0.456	0.0 ±17.8	0.358	0.0 ±13.3	0.714	0.0 ±16.8	0.467
S02	0.0 ±10.9	0.118	0.0 ±10.9	0.144	0.0 ±11.0	0.070	0.0 ±11.0	0.035
S03	0.0 ±16.1	0.242	0.0 ±16.5	0.102	0.0 ±10.2	0.786	0.0 ±10.9	0.753
S04	0.0 ±20.9	0.563	0.0 ±22.9	0.426	0.0 ±15.7	0.784	0.0 ±23.6	0.358
S05	0.0 ±17.5	0.696	0.0 ±15.3	0.778	0.0 ±24.0	0.172	0.0 ±24.0	0.167
S06	0.0 ±17.8	0.328	0.0 ±17.1	0.417	0.0 ±18.6	0.152	0.0 ±18.1	0.281
S08	0.0 ±14.9	0.720	0.0 ± 6.2	0.958	0.0 ±17.2	0.598	0.0 ±18.9	0.482
S09	0.0 ± 7.6	0.893	0.0 ± 6.0	0.935	0.0 ±15.8	0.356	0.0 ±16.5	0.235
S10	0.0 ±11.2	0.577	0.0 ±10.6	0.639	0.0 ±12.8	0.366	0.0 ±13.5	0.161
All	0.0 ±15.2	0.813	(†) 0.0 ±14.3	0.836	(†) 0.0 ±15.8	0.796	(†) 0.0 ±17.1	0.755

	(e) $tStd_G$		(f) $\Delta\sigma_H$, $\frac{\Delta\sigma_H}{\sigma_G}$		(g) $\Delta\sigma_L$, $\frac{\Delta\sigma_L}{\sigma_G}$		(h) V_T	
	ϵ_{Abs} (mL)	r (1)	ϵ_{Abs} (mL)	r (1)	ϵ_{Abs} (mL)	r (1)	ϵ_{Abs} (mL)	r (1)
S01	0.0 ±17.7	0.363	0.0 ± 6.8	0.934	0.0 ± 6.2	0.946	0.0 ± 5.1	0.963
S02	0.0 ±10.8	0.186	0.0 ± 5.7	0.855	0.0 ± 6.1	0.831	0.0 ± 4.8	0.898
S03	0.0 ± 8.9	0.844	0.0 ±11.8	0.701	0.0 ± 7.8	0.883	0.0 ±13.4	0.590
S04	0.0 ±25.2	0.069	0.0 ±13.9	0.835	0.0 ±13.9	0.834	0.0 ±15.5	0.791
S05	0.0 ±19.5	0.600	0.0 ± 9.2	0.925	0.0 ± 9.6	0.919	0.0 ± 7.3	0.954
S06	0.0 ±18.7	0.100	0.0 ±12.5	0.747	0.0 ±12.8	0.731	0.0 ±10.9	0.814
S08	0.0 ±21.4	0.093	0.0 ±14.2	0.751	0.0 ±15.0	0.719	0.0 ±11.0	0.860
S09	0.0 ±12.9	0.649	0.0 ± 5.6	0.943	0.0 ± 9.6	0.825	0.0 ± 7.1	0.907
S10	0.0 ±13.6	0.139	0.0 ± 9.3	0.738	0.0 ± 7.6	0.835	0.0 ± 7.0	0.860
All	(†) 0.0 ±16.8	0.766	0.0 ±10.4	0.917	0.0 ±10.3	0.920	0.0 ± 9.7	0.929
