# Supplementary material for: Accuracy and reliability of noninvasive stroke volume monitoring via ECG-gated 3D electrical impedance tomography in healthy volunteers
Source: PLoS One. 2018 Jan 26;13(1):e0191870. doi: 10.1371/journal.pone.0191870 (PMC5786320; doi:10.1371/journal.pone.0191870)
Supplement: S3 Table — Subject-specific and overall performance for a selection of eight features (a) to (h) and hypothesis (H3) relative SV via subject-independent calibration. The performance between ΔSVEIT and ΔSVRef is evaluated in terms of angular error ϵα and angular concordance rate CR. The (†) indicates unrealistic solutions with calibrations coefficients not having identical sign for all subjects. Cell shadings indicate whether the acceptance criteria (see methods section) are met (green), not met (red), or met but with unrealistic calibration coefficients (yellow). (PDF) [file pone.0191870.s011.pdf]

Table S3. Subject-specific and overall performance for a selection of eight features (a) to (h) and hypothesis (H3) relative SV via subject-independent calibration. The performance between ΔSV_{EIT} and ΔSV_{Ref} is evaluated in terms of angular error ϵ_α and angular concordance rate CR. The (†) indicates unrealistic solutions with calibrations coefficients *not* having identical sign for all subjects. Cell shadings indicate whether the acceptance criteria (see methods section) are met (green), not met (red), or met but with unrealistic calibration coefficients (yellow).

	(a) $\Delta\sigma_H$		(b) $tStd_H$		(c) $\Delta\sigma_L$		(d) $tStd_L$	
	ϵ_{Abs} (mL)	r (1)	ϵ_{Abs} (mL)	r (1)	ϵ_{Abs} (mL)	r (1)	ϵ_{Abs} (mL)	r (1)
S01	7.0 ± 22.0	66.7	−3.0 ± 23.8	100.0	−17.5 ± 4.5	100.0	−21.4 ± 3.7	100.0
S02	−19.5 ± 24.5	77.8	−32.9 ± 14.0	28.6	−6.2 ± 25.5	63.6	−14.1 ± 18.7	66.7
S03	−24.7 ± 8.5	80.0	−27.3 ± 10.1	60.0	−35.2 ± 6.4	20.0	−31.7 ± 6.7	60.0
S04	3.4 ± 21.5	88.9	−1.0 ± 23.9	88.9	−10.5 ± 16.0	100.0	−15.3 ± 17.2	100.0
S05	−7.0 ± 18.4	83.3	1.1 ± 19.9	100.0	−11.5 ± 22.5	80.0	−23.1 ± 12.1	50.0
S06	−18.2 ± 28.3	63.6	−10.7 ± 33.4	61.5	−7.6 ± 20.6	75.0	−19.1 ± 13.5	66.7
S08	−22.2 ± 11.5	80.0	−12.2 ± 11.5	100.0	−18.4 ± 14.4	50.0	−23.3 ± 11.5	40.0
S09	14.8 ± 15.8	80.0	18.5 ± 12.7	80.0	−16.4 ± 3.4	100.0	−16.4 ± 4.0	100.0
S10	17.9 ± 18.6	77.8	19.9 ± 17.7	66.7	−2.6 ± 24.8	75.0	2.7 ± 24.0	80.0
All	−5.3 ± 25.2	76.9	−4.9 ± 26.5	73.8	−12.1 ± 20.3	70.4	−17.4 ± 16.7	70.2

	(e) $tStd_G$		(f) $\Delta\sigma_H$, $\frac{\Delta\sigma_H}{\sigma_G}$		(g) $\Delta\sigma_L$, $\frac{\Delta\sigma_L}{\sigma_G}$		(h) V_T	
	ϵ_{Abs} (mL)	r (1)	ϵ_{Abs} (mL)	r (1)	ϵ_{Abs} (mL)	r (1)	ϵ_{Abs} (mL)	r (1)
S01	−22.8 ± 0.1	100.0	9.1 ± 8.5	100.0	9.6 ± 8.2	100.0	16.9 ± 8.1	100.0
S02	−21.1 ± 7.7	85.7	−6.4 ± 14.9	88.9	−4.3 ± 15.9	100.0	−9.3 ± 11.7	88.9
S03	−19.9 ± 7.7	100.0	−26.0 ± 2.3	100.0	−23.3 ± 3.3	100.0	−27.4 ± 7.2	40.0
S04	−23.7 ± 5.9	100.0	10.6 ± 14.0	87.5	6.4 ± 14.3	85.7	−2.7 ± 15.8	100.0
S05	−22.1 ± 11.4	50.0	−11.9 ± 4.6	100.0	0.6 ± 26.7	83.3	−15.7 ± 3.1	100.0
S06	0.6 ± 45.7	50.0	2.7 ± 20.6	83.3	5.4 ± 20.2	84.6	2.1 ± 17.7	92.3
S08	−26.0 ± 9.1	40.0	−29.3 ± 9.9	40.0	−35.0 ± 12.1	40.0	−4.0 ± 15.0	100.0
S09	−14.4 ± 2.0	100.0	0.1 ± 12.9	100.0	−3.0 ± 14.0	100.0	−10.4 ± 7.9	100.0
S10	−16.0 ± 2.0	100.0	16.1 ± 19.3	75.0	13.4 ± 20.6	71.4	21.2 ± 13.5	85.7
All	(†)−15.6 ± 25.7	73.3	−1.9 ± 20.4	83.9	−1.7 ± 21.7	84.2	−1.5 ± 18.5	89.8
